# Lectures on the Diagnosis and Treatment of Diseases of the Lungs

**Published:** 1841-01-16

**Authors:** W. W. Gerhard


### MEDICAL EXAMINER.

DEVOTED TO MEDICINE, SURGERY, AND THE COLLATERAL SCIENCES.

No. 3.] PHILADELPHIA, SATURDAY, JANUARY 16, 1841. [Vol. IV.

### LECTURES ON THE DIAGNOSIS AND TREATMENT OF DISEASES OF THE LUNGS.

BY W. W. GERHARD, M. D.

### LECTURE XIII.

#### TUBERCULOUS PHTHISIS.

Tuberculous phthisis, or consumption of the lungs, is the most formidable disease of the thorax; that is, a much greater number of individuals fall victims to it than to any other affection. It is natural, therefore, that we should study the disease with attention, and should strive to acquire the means of detecting it in that early stage when treatment is often of decided benefit. In the later stages, unfortunately, we do not possess the means of arresting the progress of the disease; we may, it is true, to a certain extent, modify the symptoms, and thus alleviate the sufferings of the patient, but we can only in a few cases contribute to positive recovery. Even in these few instances we do not possess the same controlling influence as in many other diseases, but must limit ourselves to acting strictly as the handmaid of nature, and aiding the process of cure which she institutes. It is very probable that our power of control will be greater when the intimate pathology of the disease is more thoroughly understood, and the circumstances which favour the formation of tuberculous matter are completely known.

Consumption of the lungs is frequently regarded merely as a local disorder, but although the chief lesions are seated in the pulmonary organs, the essential characters of the disease depend much more upon its diffusion through the whole body than upon the local mischief, which is comparatively slight. The cause of the fatal termination is sometimes to be found in the local lesions, and the secondary exhaustion and irritation caused by them, and at other times in the general disorder which attends both the earlier and latter stages of pulmonary consumption.

Hence consumption is to a great extent a complex disorder, and must be regarded in two distinct points of view; which must be kept steadily in mind, not only in the diagnosis but the treatment of the disease. On the one hand there is a local mischief which is often accompanied with inflammatory symptoms, and on the other there is a vice or diseased action going on in the whole economy, which is brought especially into play in the lungs, but is rarely confined to these organs. This diseased condition of the whole body has received different names; by some it is called the tuberculous diathesis or cachexia, and by others the scrofulous constitution; but when the latent mischief is brought into action, it then receives a name from the organ, which is most decidedly attacked, and the original predisposition is almost lost sight of. Hence the tuberculous diathesis, that is, the general disorder, may be developed to a very intense degree, and yet the local mischief may be slight, and tubercles may be scattered over a large number of organs. In these cases it is difficult to say whether the disease should be called pulmonary phthisis or not, for the disease of the lungs scarcely preponderates over that of the rest of the body, and the seat of the disorder is to be looked for in the fluids rather than in the solid tissues; at most the affection of the lungs is important in such cases as a sign of the general disorder, not as a disease in itself, and the only means we possess of modifying the progress of the disease, consist in such remedies as are essentially general in their nature.

In other cases the pulmonary affection either begins as the earliest point of the disorder, or it occurs very early in the disease, and the functional disturbance of the lungs becomes so considerable that it necessarily attracts a large share of attention. This is the case in a large proportion of tuberculous diseases, especially amongst adults, in whom the lungs are not only the part which in most cases is earliest attached, but it is that which is most deeply affected, and becomes the seat of the most extensive lesion. Just in proportion to the early appearance of these lesions, and to their inflammatory character, does the disease participate more in the characters of a local and less in those of a general disorder; still, the latter part of the affection must in no case be lost sight of. Even in those cases which are most inflammatory, and which differ least from pneumonia, there is sometimes more than a common inflammation, for a secretion of tuberculous matter is added to the ordinary products of inflammation, and this secretion implies a peculiarity of constitution, either congenital or acquired, in the patient. If this peculiarity did not exist, it would be an ordinary local disease, which it evidently is not, either as regards its symptoms or lesions.

The essential character then of pulmonary consumption is, that tuberculous matter should be deposited in the lungs, and the disease may begin with the local mischief, or this may take place as an evident sequel to a constitutional disorder. In both varieties, however, the constitutional mischief is present, and the evidence of this consists mainly in the formation of the tuberculous matter. It is very clear, however, that tubercles do not constitute the disease, and we must avoid falling into an error into which the exclusive study of pathological anatomy might lead us. The disease is essentially a morbid condition which either pervades for a long time the formation of tubercle, or it is more acute in its character, and is either accompanied or quickly followed by this product; the local diseases which often precede tuberculous formations being, as we have ofteen seen, merely an exciting cause.

It is now agreed to restrict the term consumption of the lungs to the cases in which there are tubercles, although it was at one time used as synonymous with all chronic diseases of the lungs, attended with emaciation, which of course included chronic bronchitis and pleurisy.

Tubercle is the same in all its essential characters, in whatever part of the body it may be formed. It consists of a white opaque or yellowish body which increases to a moderate size, rarely more than that of a large almond, and generally much smaller, when it begins to soften and is finally converted into a very thick pasty yellowish liquid, of a dull yellow colour, and and heavy but not fœtid smell. As soon as this softening takes place, the delicate cellular membrane which always encloses tubercle like other morbid products of an analogous kind, begins to assume the characters of a pus-secreting membrane, and becomes thicker; ulceration finally takes place of some portion of it, and the matter finds its way towards the exterior of the body, generally by means of a mucous tube. At first tubercles appear under several different forms, either that of a yellow opaque granulation, or of a grayish semi-transparent one; in either case they are rounded, probably from the pressure of the adjoining tissue. In other cases the tissue affected is infiltrated with a grayish semi-transparent liquid, which does not at first reveal its natural structure; little by little this disappears, and is gradually absorbed as the quantity of the new substance increases. This infiltrated tuberculous matter is not always of a grayish semi-transparent colour; in some cases it is yellow and opaque from the very commencement, but in the greater number it passes through the changes of colour just described; these are accompanied with a corresponding change in the intimate structure of tubercle; it becomes more granular, more fragile, and less perfectly animalized. But in both cases the essential constituents of tubercle are the same, consisting chiefly of albumen, with a small proportion of the salts of lime. There is, therefore, nothing peculiar in the chemical composition of tubercle, its characters depend upon its tendency to increase and finally to soften, and on the diseased condition of the whole economy which is necessary to its production. The gradual changes which occur in its structure give rise to peculiar symptoms which are secondary to the disease properly so called. Hence in the study of tuberculous diseases in general, but especially in that of the lung, we have two sets of symptoms, one being primitive, and the other secondary, and not directly so much connected with the disease as with its effects. The patient may perish from either cause.

Although in its regular progress, tuberculous matter ends in softening, and in the formation of a pus-secreting cavity, this is not a necessary or invariable consequence. In many cases the tubercle ceases to increase after it has attained a certain size, and becomes harder and drier; the earthy matter increases in quantity, and a calcareous mass is left in place of the tubercle, surrounded by a membrane; in such cases the secondary symptoms are either wanting, or are very slight. In a smaller proportion of cases, the tubercles do not even advance so far, but are actually absorbed; this fact is difficult to prove, because tubercles are not in their earliest stage susceptible of physical demonstration, but there is every reason to admit it, for patients who have laboured under the decided symptoms of commencing phthisis, have on the one hand recovered, and on the other passed into the more advanced stages of the disease. We have, however, more direct proof of the curability of tubercle. That is the evidence derived from pathological examination, and of this there is no more striking illustration than the case of a late lamented physician of this city. It is well known that he regarded himself as labouring under pulmonary consumption at an early period of life; he recovered vigorous health, lived to the age of sixty, and finally died of a disease of the kidneys. In his case there was undoubted evidence, not merely of the previous existence of phthisis, but of its absolute cure. At the summit of each lung were cicatrices and deposits of calcareous matter, proving that some portion of the tuberculous matter had passed to the state of softening, and that another portion had become dry and indurated. We learn from pathology that the more advanced tubercles are almost never met with, unless some gray granulations or incipient tubercles are found at the same time scattered amongst or around the larger tubercles; hence the inference is very conclusive, that the granulations have disappeared in those cases in which, although there are evident indications of the larger tubercles in the cicatrices and in the calcareous matter, no trace exists of the granulations. This case has probably taken place by admitting absorption.

Phthisis is therefore strictly a curable disease, but in the majority of cases it terminates fatally, at an earlier or later period. This arises not so much from the effects of the first crop of tubercles as from the successive deposit of new ones in different parts of the lung, or rather from the accompanying fever and irritation. Hence a patient rarely dies of one attack of phthisis, except it be of a very acute form.

### ANATOMICAL CHARACTERS.

These have been already described to a certain extent. As they essentially consist in the deposit and formation of tubercles, little need be added. The most frequent variety of tubercle in the lungs is that which commences by gray granulation, and gradually passes into a more developed stage; but the infiltrated tubercle is also extremely common, although rarely found alone, that is, without the gray granulations. Both of these varieties begin at the summit of the lungs in the majority of cases, and are found with nearly equal frequency on the two sides. In other cases the tubercles are formed in a different way,—that is, at the middle or lower portion of the organ, and they then begin more frequently as the formed tubercle, without being preceded by the gray granulation; this is particularly the case where the general health of the patient is much vitiated, and the fluids of the body are much altered. In the latter case the tuberculous matter is softer, and less perfectly eliminated; but it passes more rapidly through its course, and is, therefore, dependent upon a more severe form of the disease.

The exact seat of pulmonary tubercle is difficult to point out. In fact, it is not always the same. In some cases, especially the last mentioned variety, the tuberculous matter is evidently found in the mucous membrane of the bronchial tubes and small vesicles; but the gray granulations follow the usual rule upon this subject, and are formed in the cellular tissue of the lungs, as in that of the pia mater or the spleen, and are nourished by distinct vessels distributed to each granulation. These granulations, as they enlarge, press upon the neighbouring vesicles, and gradually cause their atrophy, and finally give rise to absorption of the pulmonary tissue. The cysts are formed in the lungs, or in other organs, by newly developed cellular tissue around the tubercle; this gradually thickens as the softening advances, and, as I have already stated, it is then lined by a regular pus-secreting membrane.

The process of cicatrization is nearly the same as in other cases of cavities in the lungs; as soon as the specific tuberculous matter is completely discharged, there remains merely an ordinary cyst, which either becomes continuous with the mucous membrane of the bronchi, or is filled up by the deposition of the cellular tissue.

The condition of the surrounding tissues is very various. If the case occur as a purely constitutional disorder without previous local inflammation, the tissue remains pervious to the air, and nearly healthy; but if inflammation either precedes the formation of tubercle, or follows its development, the tissue is indurated, and of many shades of colour from a light gray to a decidedly reddish tinge. At other times the tubercular matter is infiltrated through the pulmonary tissue, and gives rise to an appearance similar to that of inflammation. When the inflammation is of that kind which disposes to the formation of tubercle,—that is, when it occurs in an individual labouring under a highly developed tuberculous diathesis, the granulations are scattered abundantly through the most inflamed portion of the tissue, which, in these cases, is often nearly similar to the local congestions, or lobular apoplexy, which occurs in connection with metastatic abscess of the lung. As this variety of phthisis is not so frequent as those in which the inflammation is comparatively slight or doubtful, the appearance is by no means a very usual one; on the contrary, n most cases, the lung is vesicular, and respiration is carried on in the immediate vicinity of the tuberculous matter.

Besides the lungs, the appendages of these organs are a common seat of the tuberculous deposit; particularly in those cases in which the disease is more general or diffused in its character. These are the serous tissues and the lymphatic glands at the root of the lungs, or as they are called, bronchial glands. In children they are even more frequently the seat of tubercle than the lungs themselves, and even in adults are a common seat of this deposit though to a less extent. But the pleuræ are more important as a seat of tubercle; they are often deposited on the adherent surface of the membrane as in other cases, and are very frequently formed in the thickness of the false membranes thrown out in the pleuræ. This subject belongs, however, more properly to the accompanying inflammations of phthisis. Tubercles are, of course, not confined to the thoracic organs; on the contrary, I have shown, in the preliminary remarks, that the disease is eminently constitutional, and that, like all cachectic disorders, the development of the peculiar product in the organ primarily affected, favours its formation in other parts; and, therefore, many organs suffer from the same cause. Of these complications, the most frequent, and perhaps most important, is the formation of tubercles in the follicles of the intestinal canal. At least, this is the most important consequence of advanced tuberculous disease of the lung, for although there are other diseases of the same kind in which the mischief is more considerable, they are not simple sequelæ of phthisis; but are earlier manifestations of tuberculous disease, the lungs generally remaining healthy until the other organs are attacked, or presenting but a few scattered tubercles, which develope themselves slowly. For a more full account of the relative frequency of tubercles, I must refer to Andral's pathological anatomy. It is, however, imperfect, because it is founded upon observations made by physicians who were studying chiefly a single variety of tuberculous disease, or at least did not extend their observations to a sufficiently large number of subjects, or to a sufficient variety of age and condition.

